# Transforming Capitalism, From Top Down to Bottom Up; A Response to the Recent Commentarie

**DOI:** 10.34172/ijhpm.2023.8338

**Published:** 2023-11-26

**Authors:** Ronald Labonté

**Affiliations:** Globalization and Health Equity, Faculty of Medicine, School of Epidemiology and Public Health, University of Ottawa, Ottawa, ON, Canada

 With ever-larger swathes of the world aflame (both literally and socio-politically) the need for a now official “post-pandemic” economic transformation is glaringly apparent. I hoped my article outlining what COVID-19 had placed on offer^1^ would stimulate debate and apparently it did, although sometimes coming from different starting assumptions. I begin my response with de Soysa^2^ and close with Waitzkin,^3^ the two commentaries representing the most robust and near polar opposites. Several of the other commentaries offer complementary insights.

## Decoupling Growth From Consumption

 De Soysa’s commentary, “Austerity by Design,” has a punchy ring to it, one that he uses to cite a number of “stylized facts” to challenge many of the similarly stylized (if different) facts presented in my article. He apparently agrees with my “larger claim that many global economic and policy processes are unfair to the poor”; just as I agree with him that “increasing average wealth and health standards of the population at large is the surest path to achieving health equity” (p. 1-2). Where we part company is where he describes “degrowth” as little more than “self-imposed austerity” (p. 1). A fairly novel term, degrowth has accumulated a fair amount of critique from conventional economists and developing country activists alike, suggesting caution and careful explication in its use. Contrary to de Soysa I did not simplistically argue “degrowth as a solution to questions of poverty, health, and fairness” (p. 1), although I did question the environmental viability of capitalism’s underpinning consumption-led growth model. De Soysa appears to agree with at least some of my argument, to the point of noting that “yes, the rich should reduce consumption, and, yes, the poor must catch up with increased growth” (p. 3). But he also argues that growth for the poor “can only come from higher growth among the rich” (p. 3), thereby providing the poor with “more markets and capital” (p. 2). We still end up with an ever-expanding and environmentally unsustainable spiral of consumption, which degrowth economists argue is the real issue.

 If growth was decoupled from consumption and reflected, instead, more of the “prosperity” and “caring” measures that post-growth economists are urging, and which Meurs and colleagues describe in their commentary,^4^ there would be little to quibble about. But what de Soysa espouses is a continuation of capitalist status quo growth. He does not ignore the negative environmental externalities that can accompany growth but glosses over them with comments such as “being wealthy correlates best with local-level environmental outcomes” (p. 2). This stylized fact may be true, but it is also an outcome of colonial legacies upon which much of that wealth accumulation rested (and by some accounts, still does), and the grossly distorted global environmental footprints of the world’s richest 10% whose consumption accounts for half of CO2 emissions,^5^ five times more than the emissions produced by the world’s bottom 3.1 billion. It is this disequalizing aspect of our current growth economy that challenges any continuation of the status quo.

## Marketing Fair Growth

 The contribution from Meurs, Koutsoumpa, and Huisman, “…Words Count!,” like other commentators, found the concept of “degrowth” problematic, not so much for what it implies than how its use as a policy frame is unlikely to create the needed public health activist pressure needed for change. The authors, all affiliated with the Dutch development non-governmental organization, Wemos, argue that the term risks inducing the opposite. Despite referencing this concept in my article I share similar misgivings about marketing the term and agree with the Wemos commentators that, as a mobilizing strategy, it could alienate rather than inspire people. Their plea for hope-based messaging is a reminder that most people “relate growth to something positive, like improvements in health and well-being” (p. 2); and that degrowth can create an unhelpful cognitive dissonance, something I have encountered in reactions to the concept from political and social movement leaders in the “Global South.” As I suggested in a footnote to my article, “fair growth” may be a more marketable concept; although it is only when the meaning of these terms is made more explicit that they may become useful advocacy frames.

 Their cautionary commentary is particularly relevant in light of our rapidly worsening climate crises and the increased climate anxiety it creates. In a recent *Vox *article, Ritchie, the lead researcher for Oxford’s “Our World in Data” group chided climate pessimists, not so much for being wrong, but for repetitively voicing a nihilistic future in the expectation of scaring people into action.^6^ It does not work because it generates more resignation (hopelessness) than activism (hopefulness). Neither does what she calls “complacent optimism,” the belief that progress is inevitable if we just stick to the path we are already on, as de Soysa suggests. Changeable optimism, in which we “hold on to an edge of dissatisfaction,” is “the road to progress,” although here Ritchie relies primarily upon technological innovation and is silent on the unequal power relations that typify our dominant political economy. As a recent study that questioned the pessimistic assumptions of “decoupling” carbon emissions from gross domestic product growth noted, high rates of decoupling might be technically possible but not without transforming the structure of market economies.^7^

## A Residue of Discontent

 Jensen’s commentary, “Things That Become Visible, for a While, Can Leave a Residue,” finds hopefulness in many of the points raised in my article but accurately laments that “not for the first time, the doomsday machine,” a reference to Indian writer Arundhati Roy’s description of our pathological political economy, “appears more like an unstoppable juggernaut” (p. 1).^8^ She first draws attention to how the social inequities made stark by COVID-19’s sudden appearance quickly conflated to “a question of the unequal distribution of biomedical products.” While not unimportant, “efforts to address health inequities need to go far beyond ensuring equitable access to healthcare technologies” (p. 2). One aspect of contemporary capitalism she usefully highlights is the rise of “rentier capitalism” that enriches owners of income-generating assets (“rents”), distinct from the profits generated through the manufacture of goods. Others subsume rentier capitalism under the financialization of the global economy enabled by neoliberal deregulation, liberalized capital markets, and digital technologies.^9^ Given that the wealthy world is already consuming manufactured goods at environmentally unsustainable levels, making money from money rather than from making and selling more stuff may not be such a bad thing, except for three caveats. Jensen identifies the first (it perpetuates inequalities). Secondly, many in the developing world still need more stuff to achieve reasonable life expectancies. Thirdly, the mass accumulation of financial capital inevitably finds its way to the “real economy” of production and consumption. Jensen finds some optimism in a “spreading discontent and an increasing…awareness of the inequalities and injustices at the heart of our dominant economic system” (p. 3): the “residue” of what was made visible by the pandemic and remains very much with us.

## Giving Voice to the Residue of Discontent

 Bodini, in her commentary,^10^ suggests that this residue is most evident in the progressive role of social movements in opposing unjust political economies, and in “growing and nurturing alternative approaches to structuring society and improving health and well-being” (p. 2). While she acknowledges that the pandemic was “increasingly marked by violence against human rights defenders and representatives of social movements,” she echoes a “changeable optimism” in an increase in activist engagement at local and global levels, in which “building convergence across different social movements” (p. 3) is key to building the power needed for radical change. Schuftan^11^ makes a similar argument, although only after first recounting a list of progressive social movement failures in the sense of activists being heard but rarely being listened to. This participatory tokenism with which most activists are overly familiar is most recently evident in the inability of health activists, despite enormous efforts, to win a meaningful waiver to the Agreement on Trade-Related Intellectual Property Rights (TRIPS) agreement, an example of what Schuftan considers “a fatally flawed” United Nations (UN) system in need of “wholesale reform [that] can and only will come from below” (p. 2). Emphasizing their role as human rights “claims bearers” he suggests that in any future engagement with the flawed institutions of global governance such mobilized “PICSOs” (his acronym for “public interest civil society organizations”) must support only those “concrete measures that can be legally enforced and measured.” Given evidence suggesting that intergovernmental binding agreements (treaties) that lack enforcement measures rarely demonstrate substantive change in the issues they address (to say little of the multiplying array of non-binding UN declarations),^12^ this is a formidable challenge to which the World Health Organization’s (WHO’s) negotiating text for a pandemic treaty, despite “PICSO” calls for strong (binding) and enforceable language, has yet (as of November 2023) to rise.

## Whose Caring Economy?

 Cohen, in her supportive commentary on “The Values of a Care Economy,” makes an important point: that our current capitalist system that I suggest a “caring economy” could offset or replace already embeds care within its economics.^13^ However, it is care that exploits the social reproductive labour of women and, secondarily, racialized populations. In both instances the growth imperative of capitalism is always seeking to minimize the costs of labour “for example by clustering marginalized populations into a smaller set of gendered and racialized jobs” (p. 2). We saw that manifest in the pandemic in many ways: the disproportionate risks faced by women health care workers (comparatively underpaid to their male counterparts), personal care providers in seniors’ residences (often émigré women), and the continuing double burden of family and household care unequally borne primarily by women. As she succinctly summarizes, “The care economy is…integral to socioeconomic inequality and inequities in the capitalist political economy” and its transformation “hinges on changes to the perceived value, status, and material rewards of caring work” (p. 2). Take-home message: we need a caring rather than accumulative economy, but not one borne of capitalism’s exploitative necessities.

## Capitalism: Reform, Transform, or Overthrow?

 By title alone, Benos’ commentary, “…Questioning Capitalistic Dominance,” declares stark disagreement with de Soysa’s paean to capitalism’s beneficence.^14^ Like other commentators, Benos uses the COVID-19 pandemic to illustrate “the need to overthrow the ruling capitalist system” (p. 2), suggesting that what the different reform positions described in my article share in common an “attempt to control the aggressive greed of capital” (p. 2). In common with Waitzkin^3^ Benos contends that the pandemic’s “provisional only return of the state” reveals “the unwillingness of governments” to actually do so, a point on which I have little disagreement. What remains missing, however, is a roadmap for bringing about the demise of the capitalist hegemony apart from urging a renewed working-class activism.

 Waitzkin’s commentary, “‘Post’-pandemic Capitalism: Reform or Transform?” elaborates more fully than Benos why capitalism, if not euphemistically overthrown, must certainly be transformed and not merely reformed. He notes my own ambivalence about the possibility that some progressive reforms within capitalism could lay some foundations for, if not transforming, then at least morphing capitalism into something quite different from its present neoliberal version. He also suggests that I should have centered my arguments around a critique of capitalism, as I have done in much of my other writing.^15^ In that respect, I have little to disagree with in his characterization of capitalism’s classist structure and reliance upon racism, sexism, extractivism, and rapacious accumulation, extending even to the role of industrial agriculture in creating zoonotic risk. Elucidating these depredations was simply not the descriptive foreground of the article, the intent of which was to identify (and critique) some of the economic recovery ideas generated by the pandemic. In a longer piece, assessing capitalism’s pathogenic past and present would definitely have been the foundational background.

 Waitzkin makes one trenchant (and spot-on) criticism: My article’s silence on the contradictory nature of the capitalist state. Whether pursued as stakeholder capitalism, green growth, or degrowth/post-growth, all of the models I describe assume a rational and potentially benevolent state. Waitzkin does not dispute the positive contributions to well-being to which tax-redistributive and state-funded welfare contribute but argues that the “the main role of the capitalist state is to protect the capitalist economic system” (p. 2). Such beneficence, he points out, predictably constricts or disappears whenever capitalism experiences another of its recurrent crises, much as the public bailouts to foreclose the 2008 global financial crisis were almost immediately followed by new rounds of fiscal austerity. The failure (reluctance?) of most governments to dismantle the global banking system they chose to liberalize or to tax and regulate its financialized speculation into useful public goods now sees their use of quantitative easing fuelling inflationary asset bubbles that are (once again) increasing, rather than decreasing, wealth inequalities.

 What does Waitzkin’s invocation of a long-standing Marxist critique of the capitalist capture of the state mean for “mission economies,” a term coined by Mariana Mazzucato, an internationally influential economist, which calls on governments to be much more actively engaged in economic planning and implementation?^16^ Rather than bail out capitalist market failures as states usually do (the 2008, and now the pandemic, crises), Mazzucato argues that they should use their legislative, regulatory, and taxation authority to shape such markets to produce democratically decided-upon social and environmental outcomes. Her arguments are a refreshing tonic after four decades of neoliberal obeisance to market fundamentalism and many states’ apparent past (and in some cases still present) eagerness to privatize themselves. But can mission economies de-toxify capitalism’s fundamental logic of accumulation (via continuing spirals of production/consumption or financialized growth, however diminished in pace)? Or does it risk extending capitalism’s toxic reign by urging more participatory forms of governance and a new set of “missions” that could placate rather than transform? These are question in which, with apologies to Professor Waitzkin, I remain ambivalent in the sense of entertaining a ‘both/and’ possibility rather than accepting an “either/or” certitude.

## Top-Down or Bottom-Up?

 The WHO’s recently completed Council on the Economics of Health for All (2020-2023), an all-female group comprised of many of the world’s leading heterodox and feminist economists, appears similarly equivocal. The Council released its final report in May 2023,^17^ calling for “a new political economy based on *Health for All”* (p. v) in which “policy makers must actively create and shape an economy that delivers on goals that are critical to human and planetary wellbeing” (p. 9). Emphasizing the role of policy makers and the need to “re-invest in the ability of governments to drive transformative change” (p. 47) represent a “top-down” approach characteristic of Mazzucato’s concept of mission economies. But it still begs the questions posed above.

 On the one hand, the Council supports many of the arguments made by other progressive think tanks and civil society organizations on the need for aggressive and redistributive tax reforms at global and national scales, alongside “a redesign of the international architecture of finance” (p. 27). This overhaul includes revising the economic premises and governance structures of the International Monetary Fund and World Bank, reforms long called for by many developing countries and activist social movements. On the other hand, the Council’s final report comes close to emulating the World Economic Forum’s “stakeholder capitalism” model (p. 11) that would moderate but not transform the fundamental drivers of global markets in which small corporate monopolies or oligopolies increasingly dominate. The embrace of public-private partnerships and multistakeholder governance, even with calls for strong conditionalities to ensure equitable representation and outcomes, is correspondingly problematic.

 Waitzkin doubts the transformative potential of “top-down policies initiated by political and economic elites” (p. 3). Given the present failure of many of the world’s governments to act on climate change commitments, reverse stalling on the Sustainable Development Goals, or address worsening global inequalities his dubiety is well placed. He describes, instead, a “path toward revolutionary transformation of…capitalism” as lying in “bottom-up” local communities and “the implementation of solidarity economies, an expansion of local and regional mutual aid, a transcendence of the ‘leviathan’ that comprises the capitalist state with the construction of communal governance structures, and other creative innovations” (p. 3). In a recent book, Freudenberg similarly notes a growth in worker cooperatives and mutual aid groups following the 2008 global financial crisis, seeing in them the potential to create stronger cross-movement activism that explicitly confronts capitalism as the fundamental problem.^18^ Elsewhere I have argued that “enlarging the role of worker, producer, and consumer cooperatives is one of the feasible means to erode capitalism’s dominance of political economy” because it undermines “capitalism’s defining ethos of private accumulation” (p. 67).^19^ These are not new arguments, although the anti-capitalist tenor of them is becoming more explicit.

## Bottom-Centering

 The WHO Council, in its call for a well-being economy, also claims that “communities should lead in the transformation” (p. 24). Well-being economic policy is “bottom-up, decentralized, requires coordinated implementation, and leverages the interconnectedness of government agencies, the private sector, civil society and community activities” (p. 24). We might more accurately describe this as “bottom-centering” rather than bottom-up, since without some top-down supporting fiscal and regulatory policy reforms from (still largely capitalist-captured) states, the probability of enduring transformation is slight. Capitalism has long been accompanied by moments of disengaged resistance and efforts to create alternative forms of communal living. These important and necessary efforts, however, have rarely manifest sustained or far-reaching impact. But borrowing from Jensen’s commentary, they nonetheless may have enduring “residue” in conveying a different ethos of “economy” (from *oikonomia*, Greek for “household”), one which, as Waitzkin points out, can be found in “the prioritization of ‘buen vivir’ (living well) as a core health policy in some countries and localities of Latin America” (p. 3).

 For Freudenberg, using the USA’s original and more ambitious Green New Deal as an exemplar, this ‘bottom-centering’ arises in espousing “strategic ambiguity,” a notion similar to Ritchie’s idea of optimism that hovers on “an edge of dissatisfaction.” Strategic ambiguity, Freudenberg writes, “makes a claim that can reduce kneejerk opposition” which critics may see only as “a strategy for capitalism to save itself,” but which advocates may counter is an essential transitional base for further transformation (p. 282). In that sense calls for a more activist state and the progressive tax, fiscal, regulatory, and socio-environmental policies it could use to constrain capitalism’s predatory toxicity might be seen as an interregnum, one where reform from within can lead to revolutionary transformation from without.

 Where most commentators on my article agree (and I with them) is that none of this will happen without continued cross-movement solidarity and advocacy, and the continued articulation of alternative systems of political economy that new social leaders from the millennial generation worldwide can invoke when capitalism’s polycrisis (the concurrent and intertwined shocks of inequality, ecological collapse, and polarized politics) leaves us collectively with little other viable option.

## Ethical issues

 Not applicable.

## Competing interests

 Author declare that he has no competing interests.
